# Development of an *in vitro* Plasmodium parasite killing assay for the evaluation of cell-mediated immune responses following vaccination with pre-erythrocytic malaria vaccine candidates

**DOI:** 10.1186/1475-2875-13-S1-P14

**Published:** 2014-09-22

**Authors:** Carly M Bliss, Ahmed M Salman, Rhea J Longley, Shahid M Khan, Chris J Janse, Adrian VS Hill, Katie J Ewer

**Affiliations:** 1 Jenner Institute, Oxford, UK; 2 Leiden University Medical Centre, Leiden, The Netherlands

## Background

Vaccination against liver stage malaria antigens can induce T cell-mediated immunity to the disease [1]. A viral vector vaccination regime undergoing Phase 2b clinical testing uses chimpanzee adenovirus 63 (ChAd63) and Modified Vaccinia virus Ankara (MVA) encoding liver stage antigen Thrombospondin-Related Adhesion Protein (TRAP) fused to a malaria multi-epitope string (ME). This regime induces high frequencies of antigen-specific T cells, providing 21% sterile protection and a delay to patent parasitaemia in a further 36% of vaccinees, following controlled human *Plasmodium falciparum* malaria infection (CHMI). Monofunctional IFNg-producing CD8^+^ T cells correlate with vaccine-induced protection but the associated protective mechanisms remain unidentified [2]. Developing standardized immunological and functional assays is a research-specific aim of the WHO’s Malaria Vaccine Technology Roadmap, with emphasis on novel immunoassays for investigation of cellular products reflecting cell-mediated malaria immunity [3]. Development of an *in vitro* parasite killing assay is underway, which quantifies cell-mediated killing of Plasmodium-infected human hepatocytes and investigates the underlying functional mechanisms. Additionally, the assay aims to compliment *in vivo* CHMI studies.

## Materials and methods

Human hepatoma cell lines were infected with transgenic *P. berghei* sporozoites expressing TRAP from *P. falciparum*. Freshly separated peripheral blood mononuclear cells from partial-HLA class I matched ChAd63.MVA ME-TRAP human vaccinees were enriched for CD8^+^ T cell populations. Following hepatocyte infection, enriched CD8^+^ T cells were added and incubated overnight. Level of infectivity was measured by flow cytometry through expression of GFP under a *P. berghei* promotor. TRAP-specific killing was calculated by subtraction of non-specific killing in wild type *P. berghei*-infected hepatocytes.

## Results

Transgenic *P. berghei* sporozoites with full replacement of wild type TRAP infect human hepatoma cells at >1% frequency. Preliminary results measure a 9.5-22% TRAP-specific reduction of infected hepatocytes after addition of CD8^+^ T cells from ChAd63.MVA ME-TRAP vaccinees. Equivalently, TRAP-specific parasite killing was not detected following addition of CD8^+^ T cells from control volunteers. Conclusions Successful use of transgenic parasites and the preliminary results obtained provide proof of assay concept for this *in vitro* system. Further optimization will permit the investigation of cell-mediated parasite killing of CD8^+^ T cells from a larger number of vaccinees and extend to exploration of vaccine-induced mechanisms of protection.
